# Islet Formation during the Neonatal Development in Mice

**DOI:** 10.1371/journal.pone.0007739

**Published:** 2009-11-06

**Authors:** Kevin Miller, Abraham Kim, German Kilimnik, Junghyo Jo, Uchenna Moka, Vipul Periwal, Manami Hara

**Affiliations:** 1 Department of Medicine, The University of Chicago, Chicago, Illinois, United States of America; 2 Laboratory of Biological Modeling, National Institute of Diabetes and Digestive and Kidney Diseases, National Institutes of Health, Bethesda, Maryland, United States of America; University of Bremen, Germany

## Abstract

The islet of Langerhans is a unique micro-organ within the exocrine pancreas, which is composed of insulin-secreting beta-cells, glucagon-secreting alpha-cells, somatostatin-secreting delta-cells, pancreatic polypeptide-secreting PP cells and ghrelin-secreting epsilon-cells. Islets also contain non-endocrine cell types such as endothelial cells. However, the mechanism(s) of islet formation is poorly understood due to technical difficulties in capturing this dynamic event *in situ*. We have developed a method to monitor beta-cell proliferation and islet formation in the intact pancreas using transgenic mice in which the beta-cells are specifically tagged with a fluorescent protein. Endocrine cells proliferate contiguously, forming branched cord-like structures in both embryos and neonates. Our study has revealed long stretches of interconnected islets located along large blood vessels in the neonatal pancreas. Alpha-cells span the elongated islet-like structures, which we hypothesize represent sites of fission and facilitate the eventual formation of discrete islets. We propose that islet formation occurs by a process of fission following contiguous endocrine cell proliferation, rather than by local aggregation or fusion of isolated beta-cells and islets. Mathematical modeling of the fission process in the neonatal islet formation is also presented.

## Introduction

Insulin-secreting pancreatic beta-cells play a key role in the pathogenesis of diabetes mellitus. Autoimmune destruction of beta-cells results in type 1 diabetes. Functional loss of beta-cells leads to type 2 diabetes, which is one of the most prevalent chronic diseases worldwide. However, the beta-cell ultimately functions by forming an islet of Langerhans, which is a highly vascularized micro-organ [1], [2] consisting of various other endocrine cell types that function together to maintain normoglycemia, that include alpha-cells [3]–[6], delta-cells [3], [7], PP-cells [8], [9] and epsilon cells [10], [11]. It has been reported that cellular composition differs regionally (e.g. the dorsal versus ventral pancreas; 12–14) and the size differs among islets under normal conditions as well as disease states [15]. There is a striking plasticity of islet architecture among various species, also within the same species, and under different physiological conditions such as pregnancy, obesity and inflammation [15], [16]. Although the islet is the functional unit in the regulation of glucose homeostasis, little is known about how pancreatic islets are formed during development. The widely accepted model of formation is by the local aggregation of endocrine cells that migrate from the ductal epithelium in the late embryonic stage [17]–[20]. This aggregation model is based on observations using pancreatic tissue sections. We have previously shown that two-dimensional analysis (i.e. by thinly-cut sections) has certain limitations and could only capture part of larger structures [21], which potentially hampers deducing dynamic islet formation in the pancreas. In the fetal and newborn pancreas, endocrine progenitor cells proliferate by forming cord-like structures without distinct islet formation, and differentiation occurs within these cords. (Note that these are not the pancreatic duct; 22).

We have developed a novel method to quantify beta-cell proliferation and islet formation in the intact pancreas (without sectioning) using transgenic mice with fluorescent-tagged beta-cells combined with an automated computational analysis using a macro written for ImageJ, free software available at the NIH website. Our analyses on islet development (P1–P21) include (1) changes in individual beta-cell mass in the whole pancreas; (2) immunohistochemical analysis; and (3) mathematical modeling of islet formation. We present a new model for islet formation that accounts for the morphological transformation from embryonic endocrine cord-like structures into distinct spherical islets of various sizes observed in the adult pancreas.

## Results

### Beta-Cell Proliferation and Islet Formation in the Neonatal Pancreas

**Figure 1:**
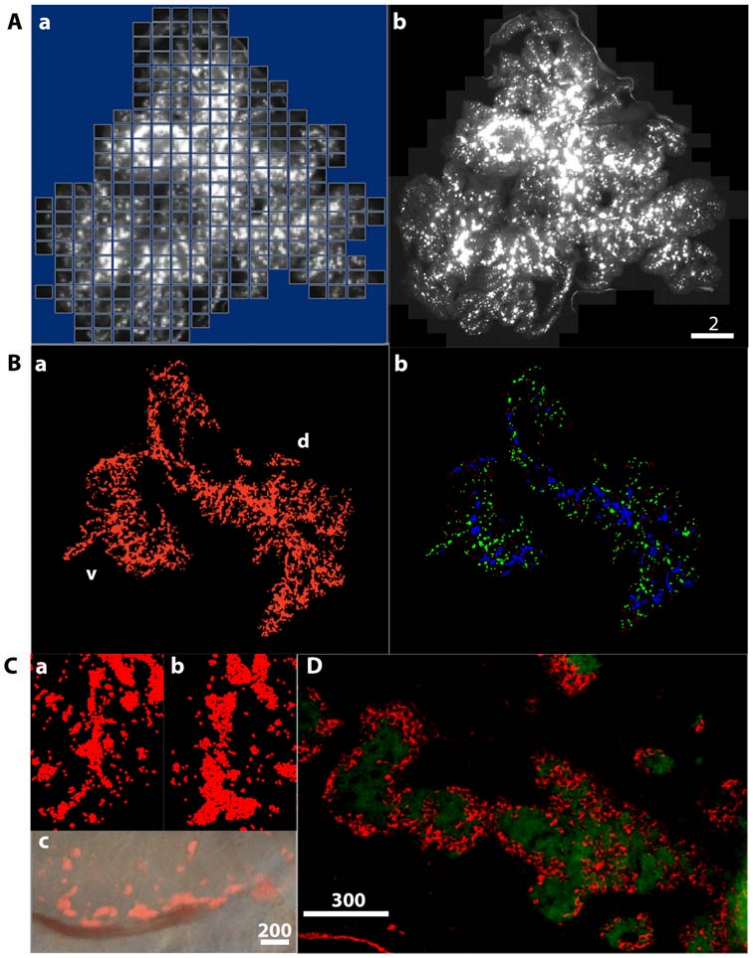
Imaging the entire distribution of beta-cells *in situ*. **A:** Virtual slice. **a.** A series of contiguous images of a specimen was taken using a 10x objective. Note a mosaic image covering the entire distribution of beta-cells. **b.** A virtual slice that combines all of the images into a single image montage. **B:** **a.** The entire distribution of beta-cells in the intact pancreas at P7 (d: dorsal, v: ventral). **b.** Three distinct population of beta-cell clusters are color-coded. Blue: interconnected islets, green: spherical shaped islets, and red: small clusters of beta-cells (<10 cells). **C:** Interconnected islets. **a.** P7; **b.** P12; **c.** A stretch of interconnected islets in the neonatal pancreas (P10). A merged image of fluorescent and bright field images of interconnected islets. Islets are often seen clustering along the large blood vessels. Scale bar is 1 mm. **D:** Immunohistochemical analysis of coalescent islets (P13). Insulin (green) and glucagon (red) staining is shown. A stretch of a continuous elongated structure with rows of alpha-cells spanning each islet-like mass is observed in the neonatal pancreas. Scale bar is 300 µm.

We have developed a method to image and quantify beta-cell distribution in the intact pancreas. RFP-expressing beta-cells were captured using the Stereo Investigator Imaging System (SI). The SI controls a XYZ-motorized stage and acquires images with spatial information. The Virtual Slice module creates a high-resolution montage composed of images obtained from multiple microscopic fields of view. Specifically, it automatically collects a series of contiguous images of a specimen and merges them into a single image montage (Fig. 1A.a), and the entire pancreas is captured as a “Virtual Slice” (Fig. 1A.b). In some pancreata, the 3D distribution of beta-cells was recorded by marking each RFP-expressing cell (Fig. 1B). The entire distribution of beta-cells at P7 shows ventral (v) and dorsal (d) pancreatic regions (Fig. 1B.a). The method has enabled us to identify stretches of interconnected islets, which are color-coded in blue together with spherical shaped islets (green) and small clusters of beta-cells (<10 cells, red) in Fig. 1B.b. A closer view of the interconnected islets is shown in Fig. 1C. Such islet-clustering is often found along large blood vessels (Fig. 1C.c). Movies that contain 3D reconstruction of the whole pancreas and interconnected islets are provided in Movie S1 online. Immunohistochemical staining of insulin and glucagon in a thin section depicts rows of alpha-cells spanning each islet-like mass within a continuous elongated structure (Fig. 1D).

### Quantification of Beta-Cell Proliferation and Islet Development

**Figure 2:**
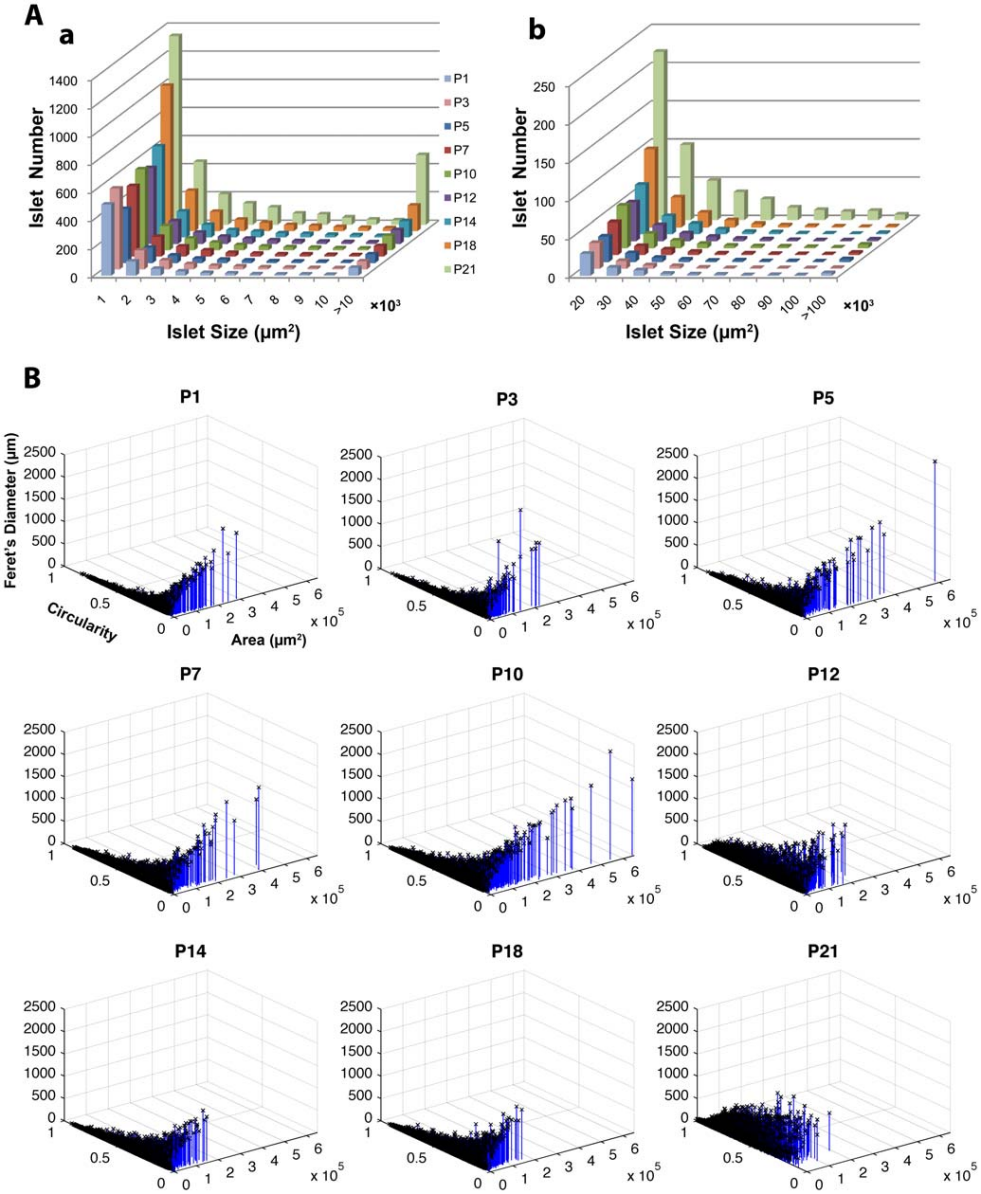
Quantification of beta-cell proliferation and islet development. **A:** A histogram showing the distribution of islets (including small clusters of beta-cells) in the neonatal pancreas (P1-P21). **a.** The distribution of total beta-cell mass is shown in the increments of 1×10^3^ µm^2^. Note that beta-cell mass larger than 10×10^3^ µm^2^ is compiled in the last column. **b.** Beta-cell mass larger than 10×10^3^ µm^2^ shown in A.a is partitioned in the increments of 10×10^3^ µm^2^. Note the difference in frequency (Y-axis) from the histogram shown in A.a. **B:** 3D scatter plot. Each dot represents a single islet/cluster with reference to size (area) and shape (circularity and Feret's diameter). Note that all plots are in the same scale.

A macro was written for ImageJ to automate the measurement of beta-cell mass, islet number, and size distribution from fluorescent images (Data S1 and Movie S2 online). The overall distribution of islets (including small clusters of beta-cells) in the neonatal pancreas (P1-P21) is shown in a histogram (Fig. 2A.a). Beta-cell mass larger than 10×10^3^ µm^2^ is further partitioned in increments of 10×10^3^ µm^2^ (Fig. 2A.b). Note that a similar size distribution is observed throughout neonatal development with increasing frequency in each bin as the animals age. Interestingly, there is a constant increase in the number of small clusters as well. Histograms with SEM and information on body and pancreas weight, total number of islets, and beta-cell mass normalized to pancreas weight at each time point are provided in Figure S1 online.

A three dimensional view of the islet/cluster distribution is shown with reference to size (area in Y-axis) and shape (circularity in X-axis and Feret's diameter in Z-axis), where each dot represents a single islet/cluster (Fig. 2B). The 3D scatter plot locates an elongated structure (e.g. interconnected islets) at a point with a greater value in area and Feret's diameter and a smaller value in circularity compared to spherical shaped islets and small clusters. All the plots are shown in the same scale so that the time course of changes in islet/cluster distribution can be recognized at a glance. There is a dynamic leftward shift, which indicates a regression in the number of elongated structures, beginning at P12 and thereafter in the developing pancreas.

### Mathematical Analysis of Islet Development

**Figure 3:**
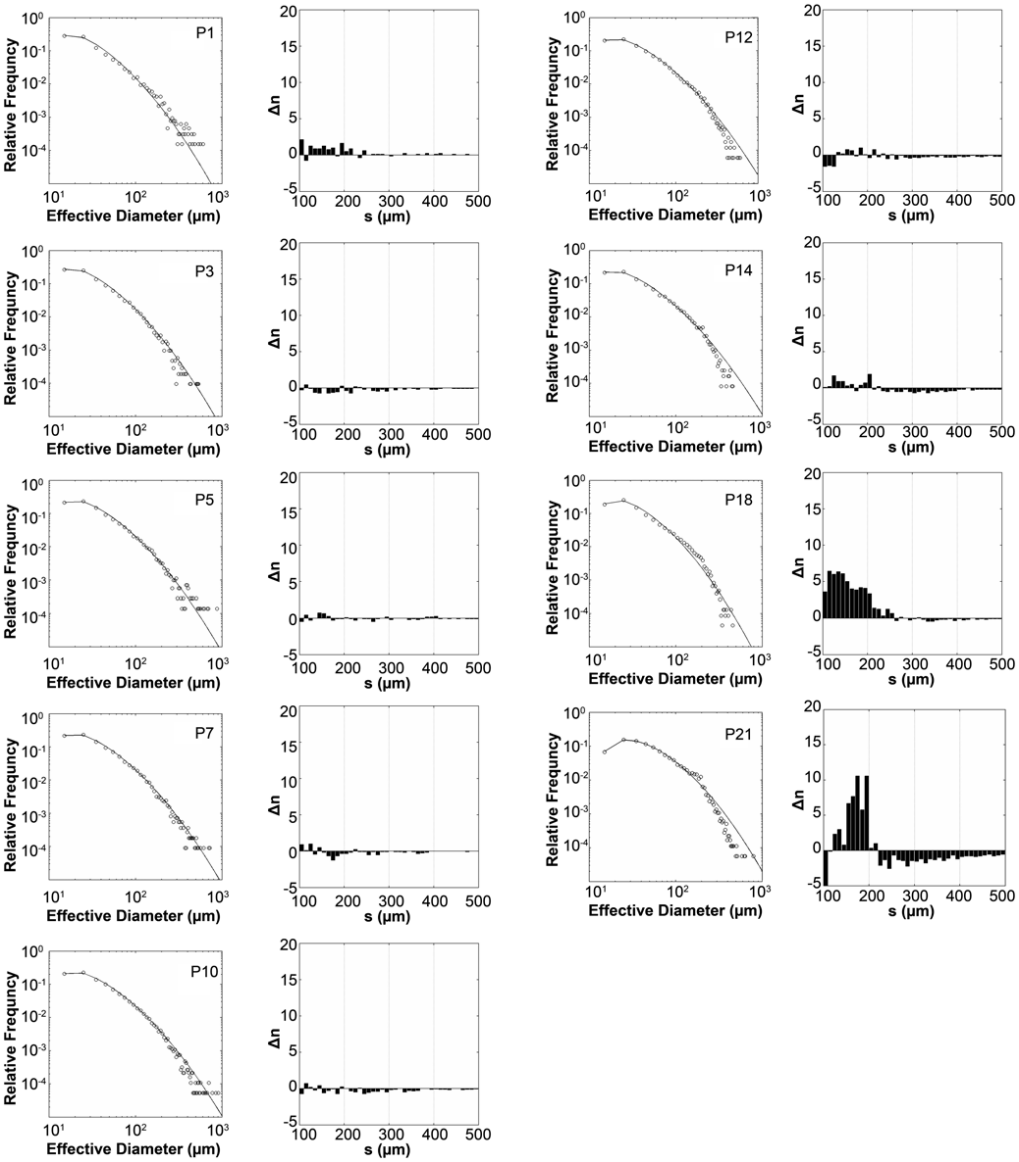
Mathematical analysis of islet development. Log plots of the distribution of beta-cell mass at each time point of the developing pancreas. The numerical value of each beta-cell mass is converted to an effective diameter, *s* (i.e. a parameter that depicts the same area of a perfect circle) and is plotted as scattered dots. Note that the overall growth of beta-cells and islet development fit into a log normal function, where at P12 and thereafter, the distribution of islets larger than 200 µm in effective diameter falls off from the curve with a leftward shift, suggesting the possible occurrence of fission events in the interconnected islet-like structures. For a clearer view, the deviation between absolute islet-size distribution and the lognormal fit, *Δn*, is shown in histograms placed right on each distribution.

**Figure 4:**
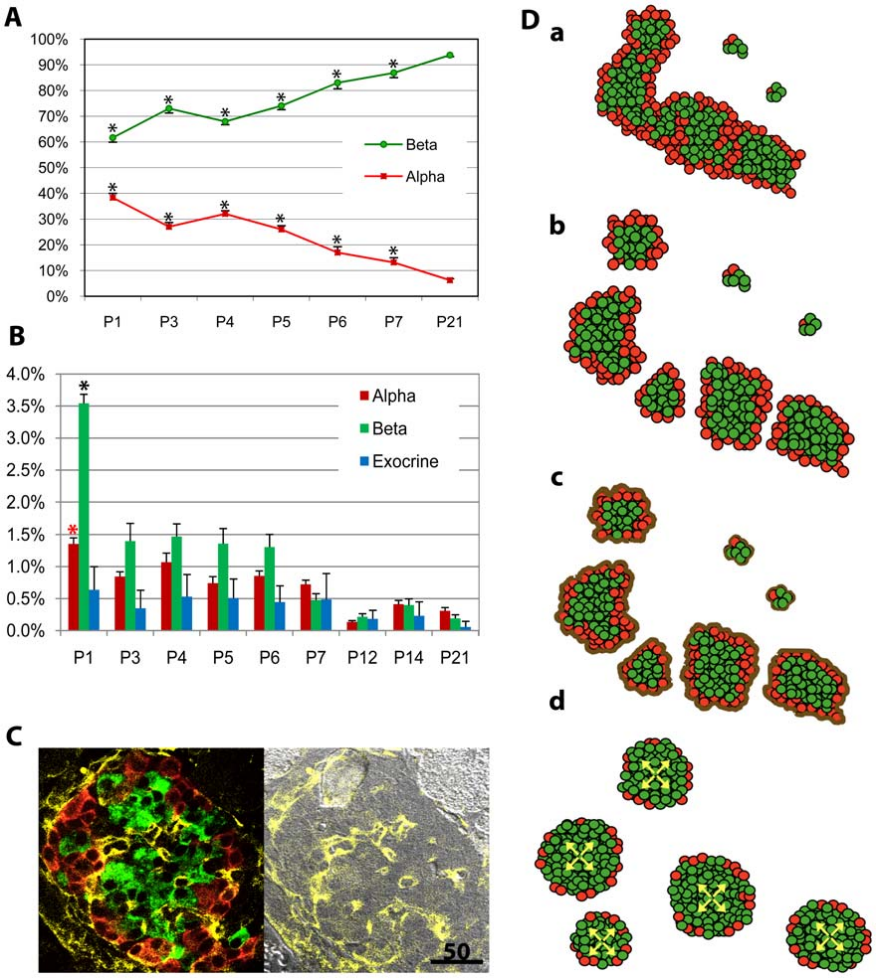
Islet formation in the neonatal pancreas. **A:** Increased ratio of alpha-cells to beta-cells in the neonatal pancreas. The difference was significant at all time points compared to the adult (8-mo) as a control. **B:** Frequency of alpha-, beta-, and exocrine-cell proliferation. Both alpha- and beta-cell proliferation at P1 was significantly increased compared to P21. **C:** Endocrine-cells coated with a layer of extracellular matrix (P1). Immunohistochemical staining for Insulin (green), glucagon (red) and collagen IV (yellow) is shown. Note that intra-islet blood vessels are also associated with the extracellular matrix. Scale bar is 50 µm. **D:** A fission model of islet formation in the neonatal pancreas. **a.** Endocrine cells proliferate contiguously, forming branching cord-like structures in the fetal and newborn pancreas. **b.** Islet formation in the neonatal pancreas may occur by fission of elongated structures composed of beta-cells and surrounding alpha-cells. The fission process appears to be random, producing islets of different size. **c.** Each islet is subsequently coated with a layer of extracellular matrix that stabilizes the structure. This process of islet formation may also result in small isolated clusters of pancreatic endocrine cells that persist even in the adult pancreas. **d.** Beta-cell mass expansion within an islet leads to an alpha-cell ratio of 5–10%.

We then employed a mathematical approach to define beta-cell proliferation and islet development in detail. When we used an “effective diameter” (defined as the diameter of a perfect disk of the same area as a given islet area) as an islet size parameter, *s*, the islet-size distribution strikingly fits the lognormal distribution (Fig. 3). Note that the shape of islet-size distributions does not change greatly with development, although total islet number increases (Table S1 for μ and σ at different postnatal days; Fig. S1B for total islet number). The invariance is a result of decreasing cell proliferation after initial rapid proliferation (Fig. 4B). Details of the derivation and the invariance of the lognormal distribution are described in the Materials and Methods section. It is of interest that no large islets (*s*>600 µm) exist after P12. Coincident with this observation, the islet-size distributions start to fall off from the lognormal curve at a certain islet size (*s*>100 µm). This result is more evident in the absolute islet-size distributions; Figure 3 shows the deviation between measured islet-size distributions and the lognormal fit. The discrepancy allowed us to interpret another islet-growth mechanism beyond the normal random growth that corresponds to the lognormal distribution. Fewer large islets (*s*>250 µm) and more small islets (100∼250 µm) than the expected number from the lognormal distribution are explained by the occurrence of islet fission, which is also supported by the morphology of large elongated islets in Fig. 1D. Islet fission may occur in large islets (*s*>250 µm at P18 and *s*>370 µm at P21) starting from around P12 to P14. The fission frequencies, calculated from the appearance of additional small islets beyond the expected number from the lognormal distribution, are 6±6 at P14; 57±9 at P18; and 166±25 at P21. Note that islet fission does not explain the enormous increase of islet number at P18 and P21, because most of this increase occurs in small beta-cell clusters (*s*<100 µm), sizes at which islet fission does not contribute significantly (Fig. S1). The increase in single cells and small clusters may reflect the cell division or differentiation of progenitor cells or neogenesis from the duct. In small islets (*s*>100 µm), however, 30 to 50% of existing small islets are a result of islet fission, while the remaining islets result from normal growth from single beta-cells.

### Islet Formation in the Neonatal Pancreas

The results from the quantification of beta-cell proliferation and islet development as well as mathematical analysis on the growth regulation has led us to propose a fission model for islet formation in the developing pancreas. An important question that arises in our model is what determines the location of cleavage points that break up the interconnected islet structure. Based on our observations of an extensive alpha-cell lining surrounding the central mass of beta-cells, we reasoned that alpha-cells might play a role in the fission of interconnected islets. The ratio of beta- and alpha-cells in the particular section shown in Fig. 1D is 51% versus 49%, respectively. We examined the time course of changes in the ratio of beta- and alpha-cells in the developing pancreas (P1–P7, P21 and 8 mo). An increased ratio of alpha-cells to beta-cells was observed at all time points compared to an adult control (8-mo; Fig. 4A; average islet number examined  = 39.6±6.1). There was also a gradual increase in the ratio of beta-cells within an islet. The frequency of cell proliferation in beta-, alpha- and exocrine-cells was measured by quantifying phospho-histone H3 staining (Fig. 4B; average islet number examined  = 66.4±6.0). Both alpha- and beta-cell proliferation at P1 was significantly higher than at P21. Acinar cells surrounding these interconnected islet structures, which expand at a high rate during development may also play a role in islet fission. A fission model of islet formation in the neonatal pancreas is detailed in Fig. 4D. Islet formation in the neonatal pancreas may occur by fission of elongated structures composed of beta-cells and surrounding alpha-cells, following the contiguous proliferation and branching of endocrine cells into cord-like structures in the fetal and newborn pancreas. The fission process appears to be random, which may explain the diversity of islet sizes seen in the adult pancreas. Islets including small clusters are coated with a layer of extracellular matrix (Fig. 4C) that stabilizes the structure. Beta-cell mass expansion within an islet leads to an increase in islet volume and the formation of spherical-shaped islets with a reduced alpha-cell ratio. This process of islet formation may also produce small isolated clusters of endocrine cells that persist throughout a lifetime.

## Discussion

A large-scale optical analysis of the entire distribution of beta-cells *in situ* enabled us to carry out quantitative studies on islet development and further mathematically model this biological event. We propose a new model of islet formation based on the following observations: (1) Endocrine progenitor cells proliferate and differentiate contiguously forming branched cord-like structures in embryos, and beta-cell differentiation occurs within these branches [21]. (2) Beta-cells proliferate contiguously in late embryos and newborns forming an identical branching pattern without forming spherical-shaped islets. (3) Distinct spherical-shaped islets are observed at P3, accompanied with elongated interconnected islet-like structures. (4) The number of such interconnected islet-like structures decreases with further pancreatic development. (5) Alpha-cell lining appears to form the putative cleavage points where islet-like structures are connected to each other. (6) Subsequent beta-cell expansion may occur within islets resulting in increased islet volume and formation of spherical-shaped (not irregular-shaped) islets. (7) Islet fission appears to occur relatively randomly, which results in various sizes of islets in the adult. (8) Mathematical analysis of a large-scale data set (collected from >100 pancreata *in situ* at 9 time points in the developing pancreas: P1–P21) shows that islet development strikingly fits a lognormal probability density function up to P10. However, beginning at P12 and thereafter, a marked leftward deviation from the lognormal distribution was observed, indicating a regression in the number of elongated structures by fission events.

Our islet fission model fills the gap between the typical epithelial cell expansion by forming cord-like structures in the embryos and the formation of spherical islets scattered throughout the exocrine pancreas in the adult. Islet fission appears to occur randomly, which may account for the different sizes of islets found in the adult, considering that beta-cells proliferate evenly [23].

The fission model also holds an important implication for possible “islet regeneration” in the adult. Xu et al. recently showed the reactivation of Ngn3 in the ductal epithelium in a surgical ductal ligation model, suggesting the existence of adult stem cells in the pancreas [24], which theoretically should further migrate out and form “a new islet” according to the aggregation model. Our model rather supports the intraislet expansion of beta-cells in the adult without new islet formation, which was first demonstrated by Dor et al. in mice under normal conditions as well as the streptozotocin-treated diabetic state [25], followed by similar observations in partial pancreatechtomy [26], [27] and ob/ob mice [28]. However, the present model nor previous publications do not exclude that the process of duct ligation [24] may initiate a neogenic process similar to that of fetal pancreas, and hence the formation of small new islets within the regenerating area. As such, islet formation by processes of delamination, migration, and aggregation may also occur.

Understanding the mechanism of islet formation is also important for the development of cell-based therapies for diabetes, such as *in vitro* differentiation of embryonic stem cells or inducible pluripotent stem (iPS) cells, since beta-cells ultimately function by forming the islet. Making beta-cells in a flat culture dish may not be sufficient (which is no doubt an important first step), but mimicking the intrinsic islet formation may require all the players (e.g. alpha-, delta-, PP- and epsilon-cells) to grow together in a three-dimensional environment.

The present study further suggests an important role of alpha-cells in beta-cell proliferation and possibly islet formation as well. We have evidence that alpha-cells at putative cleavage points for islet fission express prohormone convertase 1/3 (PC1/3), which results in local production of bioactive glucagon-like peptide 1 (GLP-1), a potent beta-cell growth factor (Hara et al., unpublished data). Note that alpha-cells in the adult only express PC2. The activation of PC1/3 is also observed in mouse models of insulin resistance such as pregnant, ob/ob and db/db mice, which may be a common mechanism in proliferating beta-cells (Hara et al., unpublished data).

In summary, the method described in the present study provides a dynamic analysis of the entire distribution of beta-cells in the intact pancreas, including small clusters of beta-cells. The quantitative and qualitative analysis of islet development and mathematical modeling of islet formation has led us to propose a fission model of the developing pancreas. An understanding of islet formation in early development may illuminate key mechanisms regulating beta-cell mass in the adult, both in states of health and disease, including the relative contributions of islet hypertrophy versus the formation of new islets. Further studies on the complex network of paracrine and autocrine interactions among endocrine cells should lead to a better understanding of beta-cell mass regulation.

## Materials and Methods

### Ethics Statement

All procedures involving mice were approved by the University of Chicago Institutional Animal Care and Use Committee.

### Mice

Mouse pancreata were excised from transgenic mice in which pancreatic beta-cells were genetically tagged with a red fluorescent protein (RFP; 21) under the control of mouse insulin I promoter (MIP). The following time course of neonatal development was studied: P1 (n = 8), P3 (n = 11), P5 (n = 11), P7 (n = 12), P10 (n = 17), P12 (n = 16), P14 (n = 10), P18 (n = 12) and P21 (n = 7).

### Preparation of Specimens

Pancreas were removed intact with surrounding tissues such as spleen and duodenum, fixed with 4% PFA at 4°C for overnight and permeabilized with 1% Triton-X 100 for two days. Specimens were further treated with saturated sucrose for several days, followed by 100% glycerol in order to clear tissue and obtain better resolution of fluorescent signals.

### Quantification of Islets/Beta-Cells in the Intact Pancreas

The entire distribution of beta-cells was captured in the intact pancreas. RFP-expressing beta-cells were visualized using the Stereo Investigator Imaging System (SI, MicroBrightField, Williston, VT). The SI controls a XYZ-motorized stage and acquires images with spatial information. The Virtual Slice module of the SI creates high-resolution montages composed of images obtained from multiple microscopic fields of view. It uses a motorized stage to automatically collect a series of contiguous images of a specimen and merge them into a single image montage. The entire pancreas was captured as “a Virtual Slice” using a 10x objective. Quantification of beta-cell mass was carried out using a macro written for ImageJ (rsb.info.nih.gov/ij/) to perform particle analysis on the virtual slices. The built-in Subtract Background command was used to remove background noise and non-fluorescing pancreatic tissue. The intensity thresholds that were used for generating a black and white mask of the islets were set automatically. This mask was analyzed using the Analyze Particle command within ImageJ. Possible artifacts (e.g. debris) smaller than one single beta-cell (<170 µm^2^; area calculated using a diameter of ∼15 µm; 29) were excluded from the analysis. Quantification included area, perimeter (a distance surrounding an area), circularity (a degree of roundness where the number 1.0 depicts a perfect circle), and Feret's diameter (the longest distance within an area) for each analyzed region. In some pancreata, three-dimensional (3D) distribution of beta-cells was recorded by marking each RFP-expressing cell manually at a higher magnification (40x), and 3D reconstruction was performed using NeuroExplore software (MicroBrightField).

### Immunohistochemistry

The mouse pancreata were excised and frozen or paraffin-embedded. Sections were stained with a polyclonal guinea pig anti-porcine insulin primary antibody (DAKO, Carpinteria, CA), a mouse monoclonal anti-human primary glucagon antibody (Sigma-Aldrich, St. Louis, MO), a polyclonal rabbit anti-phospho-histon H3 primary antibody (Chemicon, Temecula, CA), a polyclonal rabbit anti-collagen IV antibody (Abcam, Cambridge, MA), a polyclonal rabbit anti-glucagon-like peptide-1 (Abcam) and a polyclonal rabbit anti-prohormone convertase 1 (a gift from Dr. Donald Steiner). The primary antibodies were detected using different combinations of Cy2, Cy5 and Texas Red-conjugated secondary antibodies (Jackson ImmunoResearch Lab., West Grove, PA). Microscopic images were taken with an Olympus IX8 DSU spinning disk confocal microscope (Melville, NY).

### Lognormal Distribution

The lognormal distribution of islet size can be derived from the central limit theorem—a fundamental probability theorem justifying that if *k* is the sum of *n* mutually random variables, *k_1_, k_2_, …, k_n_*, then the distribution function of *k* is approximated with the normal distribution [30]. When cell-proliferation probabilities (*k_1_, k_2_, …, k_n_*) are given in discrete time intervals (*t_1_, t_2_, …, t_n_*), the total cell number in an islet increases and finally becomes (1+*k_1_*) (1+*k_2_*)… (1+*k_n_*) at time *t_n_*. Here the final cell number can be approximated as 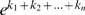 because the proliferation probabilities are small enough (*k_i_>>k_i_k_j_*). The distribution of total proliferation, *k = k_1_+k_2_+…+k_n_*, results in the normal distribution by the central limit theorem,
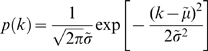
(1)


with its mean 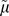 and variance 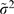. In particular, because the proliferation rate decreases rapidly after initial several divisions (see Fig. 4B), the distribution of *k* finally saturates in a certain normal distribution. Then, the normal distribution of proliferation stage *k* automatically translates into the lognormal distribution of islet diameter due to the scaling relation between total cell number and diameter of an islet. If we assume that an islet consists of single cells of the same size, the total cell number of a proliferation stage *k* is *e^k^ = (s/s_1_)^3^* where *s_1_* is the diameter of a single cell. More generally, we can describe the total cell number with a function of islet diameter: *e^k^ = a(s−s_0_)^γ^*. Here *a* is a constant and the value of the exponent γ is near 3. In particular, we have introduced a cutoff size *s_0_* because no single cells can be found below a certain size limit; we fix it with *s_0_* = 10 µm. Then, Eq. 1 becomes a lognormal distribution,
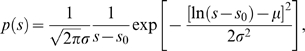
(2)where 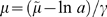 and 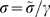.

### Statistical Analysis

Data are expressed as mean ± SEM. Statistical analyses were performed using student's *t* test. Differences were considered to be significant at *P*<0.05.

## Supporting Information

Figure S1 Neonatal development. A: Histograms shown in Fig. 2A with SEM. B: Changes in body weight, pancreas weight, total beta-cell mass, total number of islets (>500 beta-cells, which is equivalent to ∼11,000 µm^2^ based on calculation using a diameter value of 15 µm for a single beta-cell [13]), number of small beta-cell clusters (s<100 µm) and beta-cell mass normalized to pancreas weight during the neonatal development.
(1.10 MB TIF)

Data S1 The macro written for ImageJ.
(0.03 MB DOC)

Table S1 Parameter values of lognormal functions fitting islet-size distributions of postnatal pancreas and the corresponding root-mean-squares (rms) of residuals of fitting. The uncertainty of each parameter is the asymptotic standard error calculated by a fitting routine of a GNUplot package.
(1.83 MB TIF)

Movie S1 Contiguous proliferation of beta-cells. E17.5 (1 s). P0 (9 s). E17.5 Cord-like structures (20 s). The intact pancreas at P7 (32 s).. The intact pancreas at P12. Note that interconnected islet-like structures were zoomed in (80 s).
(39.68 MB MOV)

Movie S2 Screen capture of the automated macro image processing with ImageJ. An ImageJ macro, i.e. a script of instructions written for execution in ImageJ, contains the instructions for the analysis of each sample. An investigator initiates analysis by running the macro, while a virtual slice image is open (3 s). ImageJ starts parsing the macro line by line, creating a duplicate of each virtual slice sample for image processing (5 s). Duplicating the original virtual slice allows the background subtraction parameter to be optimized without having to reload the image. A threshold is automatically chosen to isolate fluorescing particles for quantification (7 s). Selected particles are reduced to a binary black and white representation (9 s). Islets and small beta-cell clusters, represented in black, are quantified. The results window on the bottom right corner displays the parameters of each particle and stores these measurements as a spreadsheet (12–26 s). The summary window appears and the window entitled “beta-1” closes when particle analysis is completed. A single virtual slice image is analyzed in under 30 seconds. Multiple images can be analyzed by embedding this image processing and analysis script into a loop syntax supported by the ImageJ macro language. The loop opens all the files in a given directory, analyzes them, and outputs results into a new location. Virtual slice background levels must be subtracted and artifacts removed prior to multiple image analysis to ensure consistency and accuracy.
(22.56 MB MOV)
