# Supplementary figures and images for: Islet Formation during the Neonatal Development in Mice
Source: PLoS One. 2009 Nov 6;4(11):e7739. doi: 10.1371/journal.pone.0007739 (PMC2770846; doi:10.1371/journal.pone.0007739)

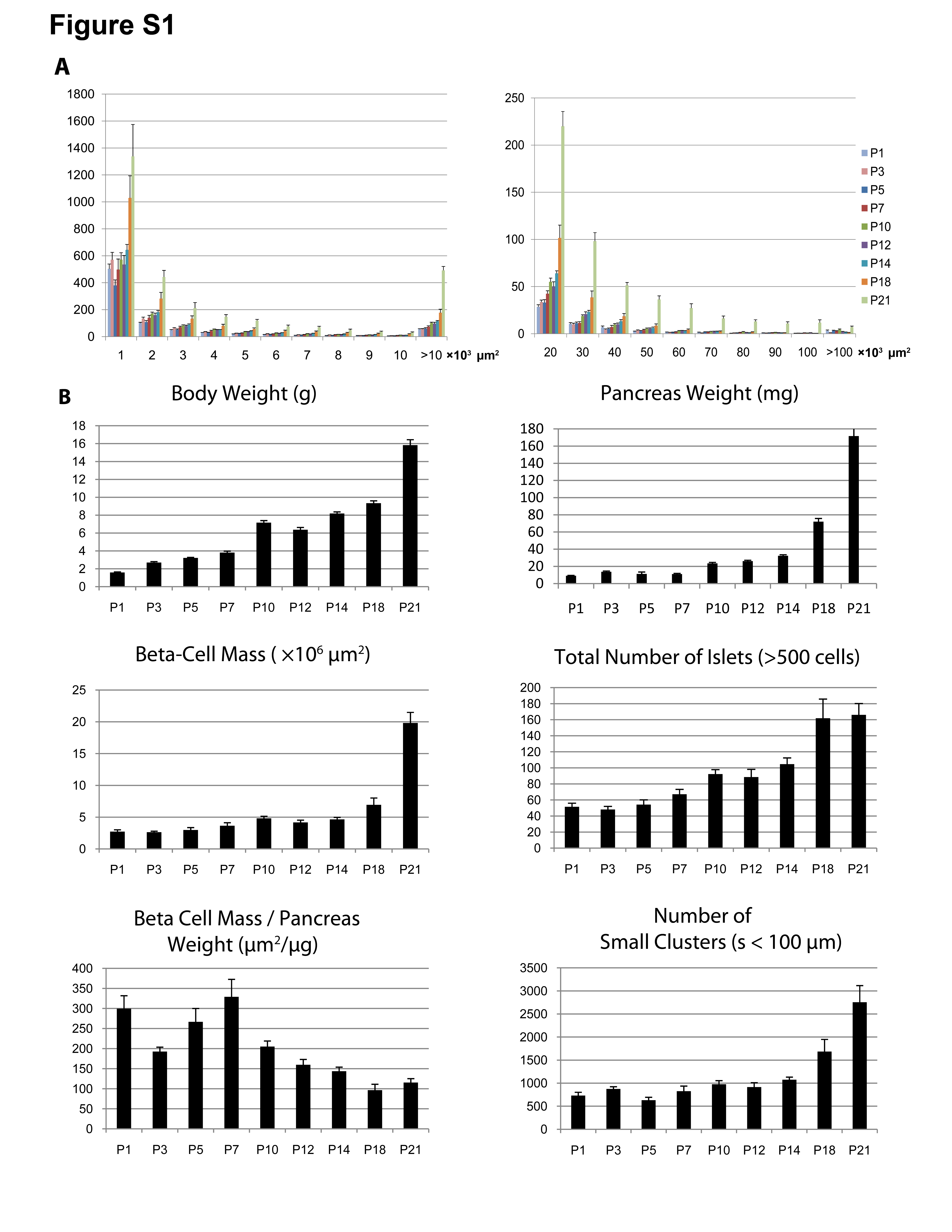

Supplement: Figure S1 — Neonatal development. A: Histograms shown in Fig. 2A with SEM. B: Changes in body weight, pancreas weight, total beta-cell mass, total number of islets (>500 beta-cells, which is equivalent to ∼11,000 µm2 based on calculation using a diameter value of 15 µm for a single beta-cell [13]), number of small beta-cell clusters (s<100 µm) and beta-cell mass normalized to pancreas weight during the neonatal development. (1.10 MB TIF) [file pone.0007739.s001.tif]
