# Supplementary material for: Islet Formation during the Neonatal Development in Mice
Source: PLoS One. 2009 Nov 6;4(11):e7739. doi: 10.1371/journal.pone.0007739 (PMC2770846; doi:10.1371/journal.pone.0007739)
Supplement: Data S1 — The macro written for ImageJ. (0.03 MB DOC) [file pone.0007739.s002.doc]

**Data S1**

//Macro to analyze particles (area > 170 micron^2) in all *.tiff files

function analyze (myDir) {

//Read myDir contents to array 'list', process images in for loop

list = getFileList(myDir);

for (i=0; i<list.length; i++){

if (endsWith(list[i],".tif")){

mkdir=myDir+list[i] + " analysis/";

//Make output directory

File.makeDirectory(mkdir);

if (!File.exists(mkdir))

exit("Unable to create directory");

print(mkdir);

//Open image

open(myDir+list[i]);

rename("original");

//Set scale and measurement parameters

run("Set Measurements...", "area perimeter circularity feret's limit redirect=None decimal=3");

run("Set Scale...", "distance=1.549 known=1 pixel=1 unit=micron global");

//Subtract autofluorescing tissue

run("Subtract Background...", "rolling=400");

//Set automatic threshold, convert to mask and perform measurements

setAutoThreshold();

getThreshold(tlow,thigh);

run("Convert to Mask");

run("Analyze Particles...", "size=170-Infinity circularity=0.00-1.00 show=[Outlines] display clear summarize");

selectWindow("Results");

saveAs("Measurements", "/home/BSDAD/mhara/ImageJ/Total.xls");

//Save output in 'myDir+list[i] + " analysis/"'.

//Note: .txt file is tab delimited

saveAs("PNG", mkdir+list[i]+"_"+tlow+".png");

saveAs("Measurements", mkdir+list[i]+tlow+".txt");

run("Clear Results");

//Clean-up

while (nImages()>0) {

selectImage(nImages());

run("Close");

}

}

}

}
